# Impact of Immunosuppressive Drugs on Patients With Percutaneous Left Atrial Appendage Occlusion

**DOI:** 10.1111/jce.70080

**Published:** 2025-08-28

**Authors:** Daisuke Togashi, Christopher R. Ellis, Zachary T. Yoneda, Jackon G. Gregory, Shunsuke Uetake, Salah H. Alahwany, Michael T. Baker, Arvindh N. Kanagasundram

**Affiliations:** ^1^ Cardiovascular Division, Department of Medicine Vanderbilt University Medical Center Nashville Tennessee USA

**Keywords:** device‐related thrombus, immunosuppressive drug, left atrial appendage closure, non‐valvular atrial fibrillation, peri‐device leak

## Abstract

**Introduction:**

Percutaneous left atrial appendage closure (LAAC) reduces the risk of thromboembolic stroke in patients with non‐valvular atrial fibrillation who cannot tolerate long‐term oral anticoagulation. However, outcomes after LAAC in patients requiring chronic immunosuppressive therapy (IMS) remain unknown. This study aimed to investigate the perioperative and long‐term outcomes of percutaneous LAAC in patients receiving chronic IMS.

**Methods and Results:**

Patients who underwent LAAC were retrospectively evaluated according to the presence of long‐term IMS therapy. Perioperative complications, clinical outcomes, and device follow‐up characteristics were evaluated. From November 2017 to March 2023, a total of 1172 patients who underwent LAAC were included (69.3% male, 74.8 years), of whom 74 were taking IMS. The most common reasons for IMS use were rheumatoid arthritis (50.0%), followed by kidney transplantation (17.6%). There were no significant differences in intraoperative complications between the IMS group and the non‐IMS group. (1.4% vs. 1.3%, *p* = 0.943). During 617 [IQR: 373–1046] days, the incidence of systemic infections was higher in the IMS group (25.0% vs. 6.1%, *p* < 0.001). There were no significant differences in peri‐device leak (PDL) or device‐related thrombus, but the IMS group had a lower percentage of patients with improvement of PDL during follow‐up (8.3% vs. 50.0%, *p* = 0.003).

**Conclusion:**

Successful LAAC in patients on IMS was achieved without increasing perioperative complications. Patients on chronic IMS did have an increased occurrence of systemic infections during long‐term follow‐up. The presence of IMS use was negatively associated with PDL improvement during follow‐up.

## Graphical abstract

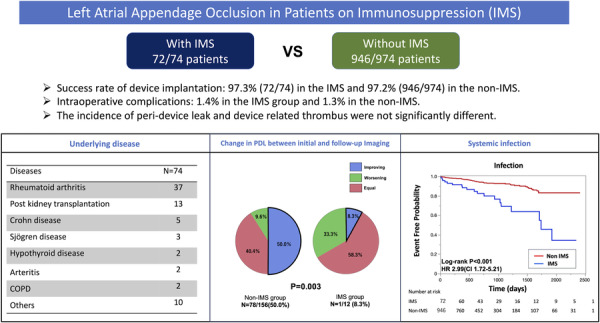

**Abbreviations**
CT: computed tomography
DAPT: dual antiplatelet therapy
DOAC: direct oral anticoagulant
DRT: device‐related thrombus
ICE: intracardiac echocardiography
IMS: immunosuppressive drug
LAAC: left atrial appendage closure
PDL: peri‐device leak
SAPT: single antiplatelet therapy
TEE: transesophageal echocardiography

## 1 Introduction

Left atrial appendage closure (LAAC) is established to reduce thromboembolic stroke risk in patients with non‐valvular atrial fibrillation with high bleeding risk [1, 2]. Patients who benefit from this therapy have a wide array of underlying conditions, including those that require chronic immunosuppressive drugs (IMS), such as autoimmune diseases or post‐solid organ transplantation. Chronic use of IMS is known to increase susceptibility to infections and raise the risk of bleeding [3, 4, 5], along with causing tissue fragility due to protein synthesis inhibition [6, 7]. LAAC carries known perioperative complications and is associated with an increased risk of bleeding, with antithrombotic medications being required for stroke prevention until the device surface is endothelialized [2, 8, 9]. The safety and efficacy of LAAC in patients on chronic IMS is not known, with no studies to our knowledge in this patient population. We sought to investigate the perioperative risk profile and long‐term clinical outcomes of LAAC in patients on chronic IMS.

## 2 Methods

### 2.1 Study Population

The IMS cohort and control cases were identified from 1172 consecutive LAAC cases performed at Vanderbilt University Medical Center, enrolled in the VaLAAR (Vanderbilt LAA Registry) registry from November 2017 to March 2023. Among these patients, the procedure was aborted in 19 cases due to a thrombus in the LAA or unsuitable LAA morphology. Additionally, 10 cases underwent surgical procedures due to LAA anatomy unsuitable for an endocardial device, 53 cases had a percutaneous LAA ligation procedure, 32 cases required reoperation due to peri‐device leak (PDL), and 11 cases were excluded because they used an investigational device (Conformal IDE). Of the remaining 1047 cases, these were divided into 74 with IMS and 973 without chronic IMS exposure.

### 2.2 Procedure of Left Atrial Appendage Closure

LAAC procedures were performed under general anesthesia guided by intracardiac echocardiography (ICE) or transesophageal echocardiography (TEE). After ultrasound guided right femoral vein access and insertion of a large introducer sheath (Cook 16 or 18Fr Check Flow), a weight‐based heparin bolus was administered and maintained during the ablation procedure to ensure a target‐activated clotting time of 300–350 s. Trans‐septal puncture was performed under fluoroscopic and TEE, or ICE guidance. All procedures were conducted with patients on uninterrupted anticoagulation therapy. LAAC devices, including Watchman 2.5 [1] and FLX(Boston Scientific, Natick, Massachusetts) [10], and Amulet (St. Jude Medical, St. Paul, Minnesota) [11], were selected by the operator based on the LAA morphology and measurements determined by preoperative imaging. Device position at the time of the implant was assessed with ICE or TEE, as well as LAA angiography and fluoroscopy. Tension or tug testing was performed to confirm device stability.

### 2.3 Postoperative Endpoints

The primary safety outcome was defined as any perioperative complication, including bleeding, stroke, or device migration. Additionally, the incidence of stroke, bleeding, all‐cause mortality, and infections requiring hospitalization was assessed during the follow‐up period. Stroke was defined as a new neurological deficit lasting more than 24 h, confirmed by imaging as either ischemic or hemorrhagic. Bleeding was defined as a clinically significant event, including a hemoglobin drop of ≥ 2 g/dL, the need for transfusion, fatal cases, or intracranial hemorrhage. Infection was defined as a systemic infection requiring hospitalization for treatment.

### 2.4 Device Follow‐Up

Device assessments were performed using TEE or computed tomography (CT). The first follow‐up was conducted at 45 or 90 days after the procedure for Amulet, Watchman 2.5 and FLX, and again at 1 year for the second follow‐up largely by TEE with CT utilized at the physician's discretion. Follow‐up imaging assessed adequate device compression, position, device‐related thrombus (DRT), and residual peri‐device leak. Residual leaks consisted of PDLs and fabric leaks, with PDLs being categorized as major ( > 5 mm), moderate (3–5 mm), and minor ( < 3 mm). Furthermore, changes in PDL were categorized as improved, unchanged, or worsened between the first and second follow‐up TEEs. The mechanisms of residual leaks were classified based on previous reports [12, 13]. The ostial line was defined as a line perpendicular to the long axis of the LAA and drawn from the left circumflex coronary artery to its opposite side. Off‐axis implants were defined as being tilted more than 30° with respect to a perpendicular line from the ostium to the long axis. Edge leaks were defined as leakage occurring despite proper placement due to insufficient compression or a discrepancy between the shape of the ostium and the device. The position of the Amulet was considered appropriate if located 10–15 mm distal to the LAA ostium. It is regarded as too proximal if the distal part of the lobe is less than 10 mm from the LAA ostium, and too distal if the proximal part exceeds 15 mm from the LAA ostium.

### 2.5 Statistics

Continuous variables are presented as mean ± standard deviation values or median (interquartile range [IQR]) values and were compared using Student's *t*‐test or the Wilcoxon rank‐sum test depending on the variable's distribution. Categorical values are presented as counts and percentages and were compared using Fisher's exact test. Events related to the incidence of stroke, bleeding, all‐cause mortality, and systemic infections requiring hospitalization were evaluated for each group using the Kaplan–Meier method. The multivariate Cox regression analysis assessed the occurrence of infections. Statistical significance was considered achieved at *p *< 0.05, and analysis was performed using JMP 18 (SAS Institute, Cary, NC, USA).

## 3 Results

### 3.1 Baseline Demographics

**Figure 1:**
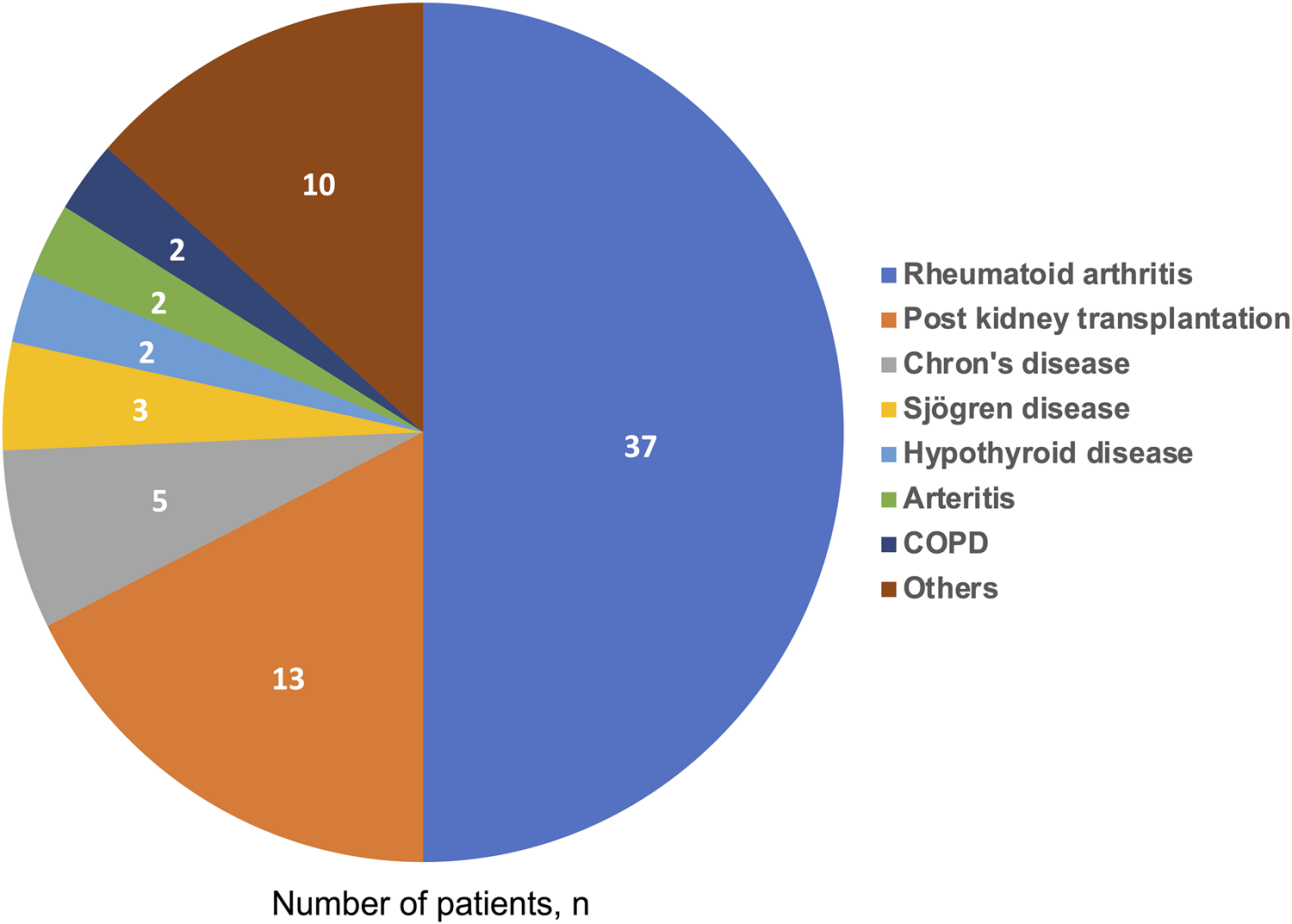
Underlying disease of patients in the IMS group. Among patients taking IMS, those with rheumatoid arthritis were the most common (50%), followed by patients after kidney transplantation (18%). COPD = chronic obstructive pulmonary disease, IMS = immunosuppressive drug.

The most common condition of patients taking IMS was rheumatoid arthritis, followed by kidney transplant (Figure 1). In the IMS group, the median daily dosage of prednisone was 5.0 mg [IQR: 5.0–7.5 mg], while methotrexate, the most frequently used non‐prednisone medication, had a median weekly dosage of 15.0 mg [IQR: 7.5–19.0 mg] (Supporting Information S1: Table 1). A total of 1047 patients had a mean age of 74.8 years, with 69.3% being male (Table 1). There were no significant differences between the IMS and non‐IMS groups in CHA₂DS₂‐VASc scores (both 4 [IQR: 4–5], *p* = 0.878) or HAS‐BLED scores (both 3 [IQR: 3–3], *p* = 0.857).

**Table 1:** Baseline characteristics.

Variable	IMS group *N* = 74	Non‐IMS group *N* = 973	*p* value
Male	49 (66.2)	677 (69.6)	0.594
Age, years	73.6 ± 7.7	74.8 ± 8.1	0.233
Body mass index, kg/m^2^	27.5 ± 5.2	28.5 ± 5.5	0.113
Smoking	43 (58.1)	574 (59.0)	0.872
Hypertension	69 (93.2)	928 (95.4)	0.432
Diabetes mellitus	48 (64.9)	574 (59.0)	0.318
COPD	17 (23.0)	204 (21.0)	0.686
Coronary artery disease	26 (35.1)	306 (31.5)	0.515
Cardiomyopathy	12 (16.2)	161 (16.5)	0.933
NYHA Ⅲ or Ⅳ	3 (4.1)	45 (4.6)	0.818
Cancer	14 (18.9)	130 (13.4)	0.200
Type of atrial fibrillation			0.788
Paroxysmal	29 (39.2)	406 (41.7)	
Persistent	35 (47.3)	440 (45.2)	
Longstanding	8 (10.8)	78 (8.0)	
Atrial flutter	2 (2.7)	39 (4.0)	
Prior ablation	6 (8.1)	96 (9.9)	0.614
Prior stroke	10 (13.5)	191 (19.6)	0.242
Embolic stroke	3 (4.1)	76 (7.8)	
Others	7 (9.5)	115 (11.8)	
Hemorrhagic stroke	2 (2.7)	82 (8.4)	0.109
CHA_2_DS_2_‐VASc score	4 [4–5]	4 [4–5]	0.878
Bleeding	29 (39.2)	338 (34.7)	0.560
HASBLED score	3 [3–3]	3 [3–3]	0.857
Prior CIED implantation	16 (21.6)	212 (21.8)	0.360
Hemoglobin, g/dL	12.7 [11.1–13.9]	13 [11.4–14.3]	0.183
eGFR, mL/min/1.73 m^2^	59 [41–65]	57 [43–69.1]	0.826
LVEF, %	55 [50–60]	60 [50–60]	0.267

*Note:* Continuous variables are displayed as the median [Q1–Q3].

Categorical variables are presented as mean ± standard deviation or *n* (%).

Abbreviations: CIED = cardiac implantable electrical device, COPD = chronic obstructive pulmonary disease, eGFR = estimated glomerular filtration rate, IMS = immunosuppressive drug, LVEF = left ventricular ejection fraction.

### 3.2 Procedural Characteristics and Outcomes

The acute success rate of LAAC device placement was 97.3% in the IMS group and 97.2% in the non‐IMS group (Table 2). The two groups had no statistically significant differences in LAA morphology or device type distribution. Perioperative complications were comparable between the IMS and non‐IMS groups, with incidence rates of 1.4% and 1.3%, respectively (*p* = 0.943). Cardiac tamponade occurred in one case in the IMS group and three cases in the non‐IMS group, with surgical repair required in one case from the IMS group. Access site‐related complications were observed only in the non‐IMS group, including one case each of arteriovenous fistula, pseudoaneurysm, and re‐bleeding. Within 30 days, there was one death due to sepsis in the IMS group and two cardiovascular‐related deaths in the non‐IMS group. Overall, the two groups had no significant difference in the incidence of complications.

**Table 2:** Procedural characteristics.

Variable	IMS group *N* = 74	Non‐IMS group *N* = 973	*p* value
Successful procedure	72 (97.3)	946 (97.2)	0.895
Procedure time, min	37 [35–50]	39 [28–52]	0.873
Fluoroscopic time, min	9.0 [6.6–13.2]	7.4 [5–10.5]	0.068
Contrast, mL	33 [20–45]	30 [20–42]	0.195
Combined procedure	6 (8.1)	105 (10.8)	0.454
Ablation	3 (4.1)	95 (9.8)	0.070
CIED implantation	1 (1.4)	11 (1.1)	0.867
Others	0 (0)	2 (0.2)	0.588
Maximum LAA ostium, mm	21.0 [18.6–23]	20.6 [18.0–23.2]	0.832
Maximum LAA depth, mm	27.8 [22.9–32.8]	28.0 [24.0–30.4]	0.725
Morphology of LAA			0.223
Cauliflower	23 (31.1)	223 (22.9)	
Chicken wing	12 (16.2)	193 (19.8)	
Windsock	34 (45.9)	531 (54.6)	
Cactus	5 (6.8)	26 (2.7)	
Procedure complication	1 (1.4)	13 (1.3)	0.943
Pericardial effusion	1 (1.4)	7 (0.7)	0.584
Tamponade	1 (1.4)	3 (0.3)	0.264
Pericardiocentesis	0 (0)	3 (0.3)	
Thoracotomy	1 (1.4)	0 (0)	
Access site complication	0 (0)	3 (0.3)	0.500
Others	0 (0)	2 (0.2)	
30‐day adverse event	1 (1.4)	18 (1.8)	0.532
Death	1 (1.4)	2 (0.2)	
Bleeding	0 (0)	5 (0.5)	
Heart failure	0 (0)	5 (0.5)	
Stroke	0 (0)	3 (0.3)	
Others	0 (0)	3 (0.3)	
Type of device^a^			
Watchman 2.5	28 (38.9)	297 (31.4)	0.196
Watchman FLX	28 (38.9)	465 (49.2)	0.091
Amulet	16 (22.2)	184 (19.5)	0.575

*Note:* Continuous variables are displayed as the median [Q1–Q3].

Categorical variables are presented as mean ± standard deviation or *n* (%).

^a^ The proportion of devices represents the percentage among successful cases.

Abbreviations: CIED = cardiac implantable electrical device, IMS = immunosuppressive drug, LAA = left atrial appendage.

### 3.3 Breakdown of Antithrombotic Medications

**Figure 2:**
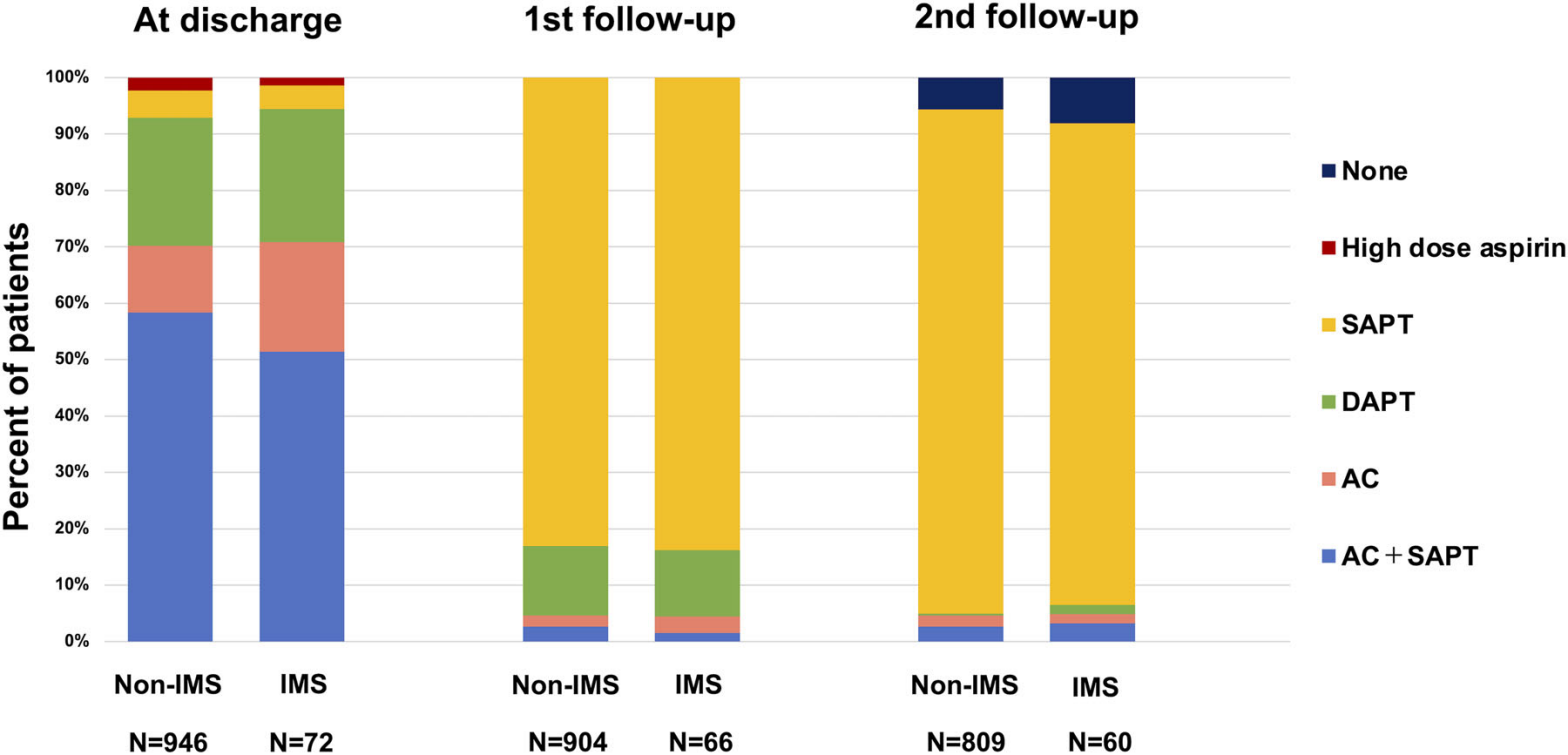
Transition of antithrombotic medications. The regimens of the antithrombotic drug in each timing is shown. AC indicates direct oral anticoagulant drug or vitamin K antagonist. AC = anticoagulant therapy, DAPT = dual antiplatelet therapy, IMS = immunosuppressive drug, SAPT = single antiplatelet therapy.

Figure 2 summarizes antithrombotic medications. The most commonly prescribed therapies at discharge were anticoagulants and single antiplatelet therapy (SAPT) (58.4%; non‐IMS, 51.4%; IMS). Of these, direct oral anticoagulants (DOACs) were most commonly used (non‐IMS; 48.1%, IMS; 40.3%), followed by dual agent platelet therapy (DAPT) (non‐IMS; 23.6%, IMS; 22.7%). Patients in the Watchman group were more frequently prescribed DOAC and SAPT post‐implant, whereas those with the Amulet device had a higher prevalence of DAPT. During the first follow‐up, if no significant events related to DRT or PDL were observed, the medication regimen was transitioned to SAPT (Non‐IMS; 83.1%, IMS; 83.8%).

### 3.4 Incidence of Device Events

**Figure 3:**
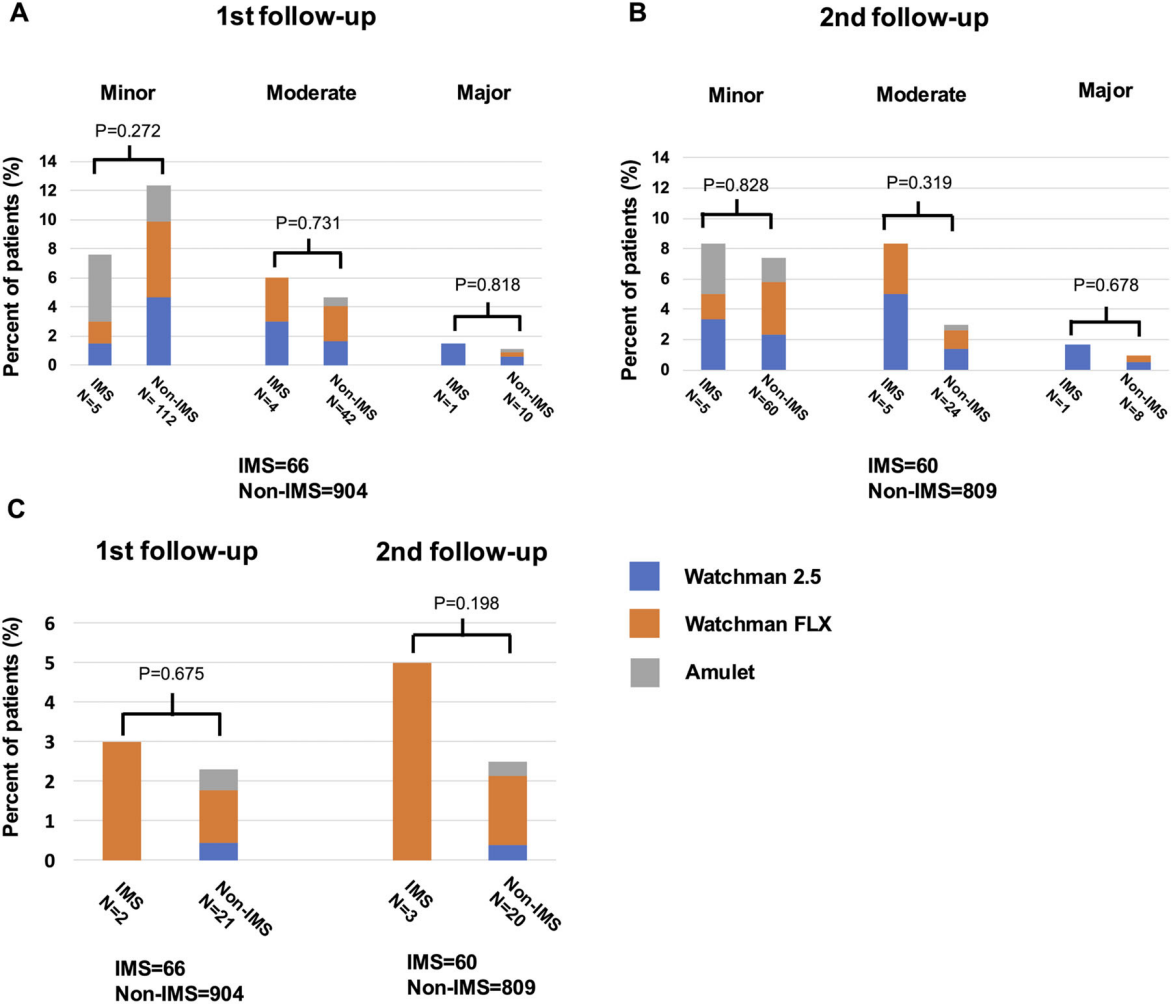
PDL and DRT at follow‐up periods. PDL at the (A) 1st follow‐up and (B) 2nd follow‐up, with no significant difference between the two groups at either period. (C) DRT at follow‐up, with no significant difference observed between the groups. DRT = device‐related thrombus, IMS = immunosuppressive drug, PDL = peri‐device leak.

**Figure 4:**
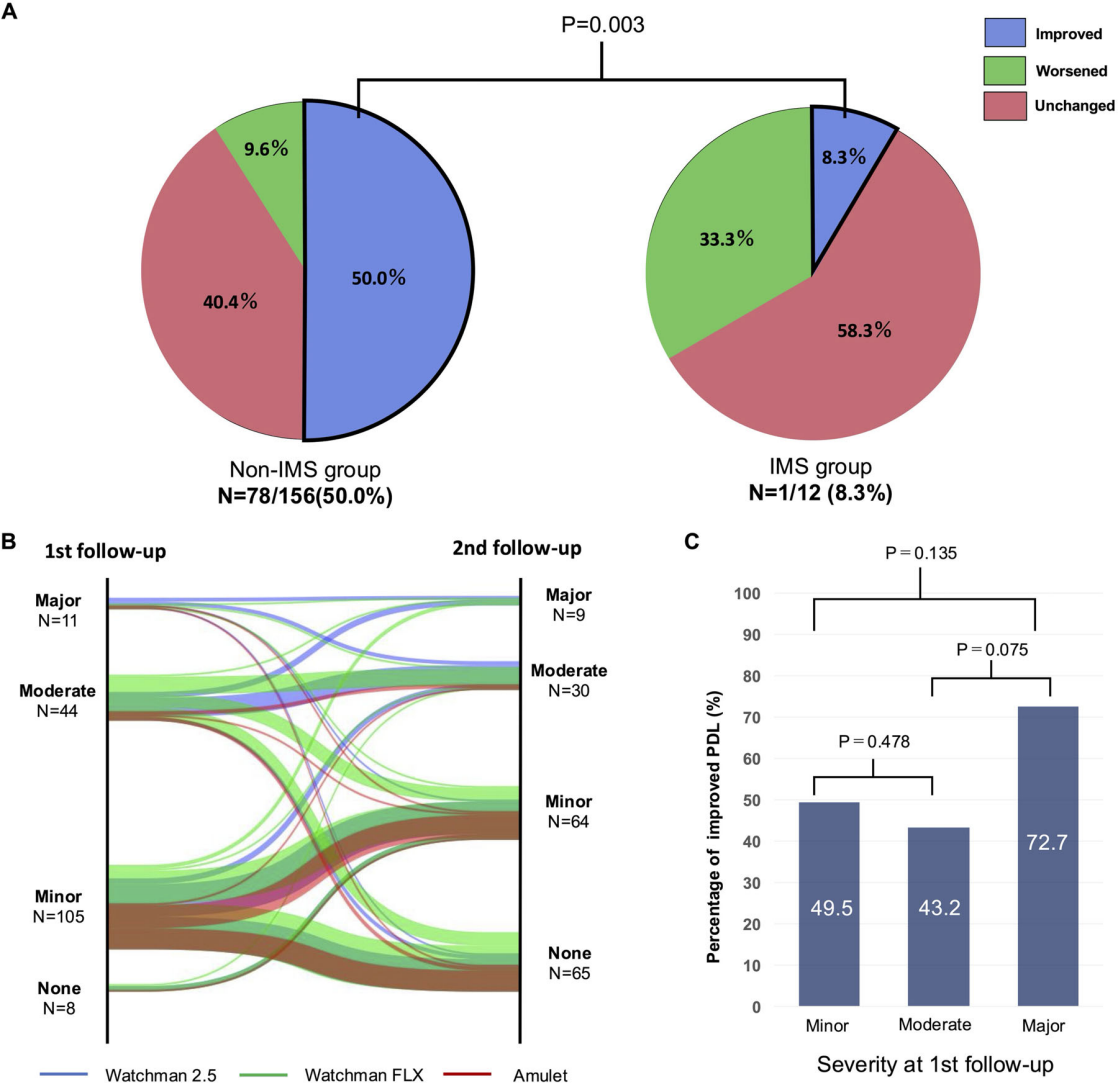
PDL changes. (A) Temporal PDL changes with or without IMS, (B) parallel plot of PDL changes by severity at the first and second follow‐up. (C) Percentage of patients with PDL improvement according to severity at the first follow‐up. Among the PDL changes, the IMS group showed significantly less improvement than the non‐IMS group (*p* = 0.003). There was no significant difference in the PDL improvement rate according to severity between the groups. IMS = immunosuppressive drug, PDL = peri‐device leak.

Figure 3 shows the incidence of DRT and PDL. At the first follow‐up, imaging was conducted for 66 patients (91.7%) in the IMS group and 904 patients (95.6%) in the non‐IMS group (TEE: 832, CT: 138). At the second follow‐up, imaging was performed for 60 (83.3%) in the IMS group and 809 patients (85.5%) in the non‐IMS group (TEE: 747, CT: 122). At the first follow‐up, PDL was detected in 15.2% of the IMS group and 18.4% of the non‐IMS group (*p *= 0.657) (Figure 3A). At the second follow‐up, PDL was observed in 18.3% of the IMS group and 11.4% of the non‐IMS group (*p *= 0.298) (Figure 3B). DRT was identified in 3.0% of the IMS group and 2.3% of the non‐IMS group (*p *= 0.675) at the first follow‐up and in 5.0% of the IMS group and 2.5% of the non‐IMS group (*p *= 0.198) at the second follow‐up (Figure 3C). Improvement in PDL between first and second imaging measurements was significantly higher in the non‐IMS group compared with the IMS group (50.0% vs. 8.3%, *p* = 0.003) (Figure 4A). Additionally, a comparison between the group with improved PDL and the group with worsened or unchanged PDL showed no association with device type, LAA morphology, or postimplantation stroke incidence (Supporting Information S1: Table 2). Similarly, no correlation was found when comparing the improvement based on the severity of PDL at the initial follow‐up (Figure 4B,C). Among the 11 cases in which PDL did not improve in the IMS group, nine patients were treated with prednisone alone, and the remaining two were treated with either tacrolimus or sirolimus in combination with prednisone.

**Figure 5:**
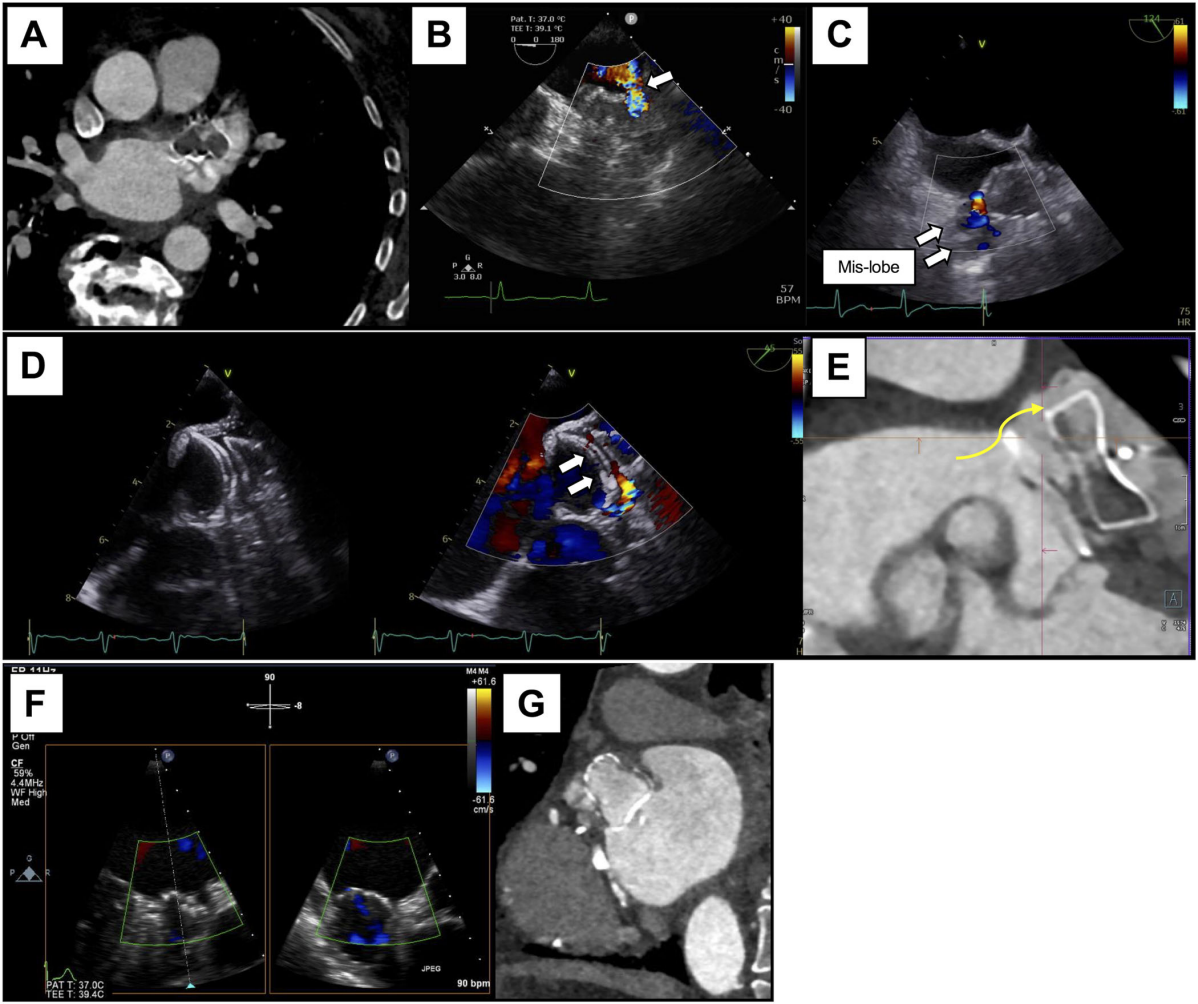
Typical images of residual leaks related to the device. (A) Off‐axis, (B) leak from the ridge side, (C) leak due to mis‐lobed, (D) leak due to disk distortion, (E) leak to the distal disk. Fabric leaks from the Watchman device can be observed in (F) transesophageal echocardiography and (G) computed tomography.

The mechanism of the leak was evaluated for each device type (Supporting Information S1: Table 3), with representative images of residual leaks shown in Figure 5. In the Watchman group, edge leaks were the most common, occurring in approximately 40% of PDL cases in both groups. Proximal placement or leaks from the ridge were observed in 25.8% of Watchman 2.5 cases and 16.3% of Watchman FLX cases. Additionally, uncovering due to pectinate muscles resulted in leaks in 21.2% of Watchman 2.5 cases and 18.8% of Watchman FLX cases. Fabric leaks due to incomplete endothelialization were observed in approximately five cases in the Watchman 2.5 and FLX groups. In the Amulet group, among PDL cases, leaks between the proximal and distal discs, as well as leaks into the distal LAA, were observed in 48.5% of cases.

### 3.5 Clinical Outcomes During Follow‐Up

**Figure 6:**
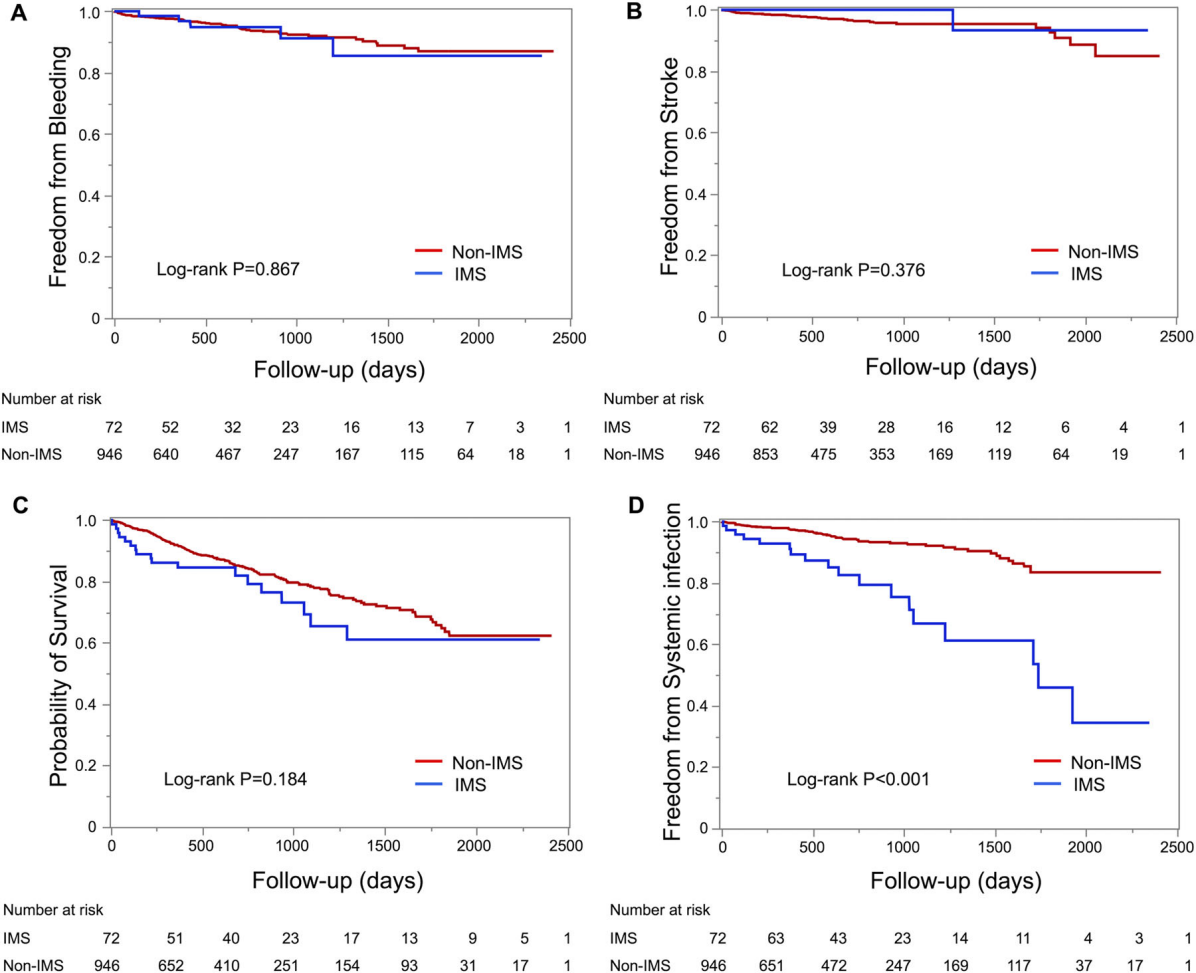
Kalan–Meier curves for clinical outcomes. Kaplan–Meier curves for (A) bleeding events, (B) stroke events, (C) all‐cause mortality, and (D) systemic infections. IMS = immunosuppressive drug.

During the median follow‐up period of 617 days [IQR: 373–1046 days], there were no significant differences in clinical outcomes between the IMS and non‐IMS groups regarding bleeding (9.7% vs. 5.8%, *p* = 0.213) (Figure 6A), stroke (1.4% vs. 3.6%, *p* = 0.266) (Figure 6B), and all‐cause mortality (25.0% vs. 17.7%, *p* = 0.134) (Figure 6C). However, infections requiring hospitalization were significantly more frequent in the IMS group compared to the non‐IMS group (25.0% vs. 6.1%, *p* < 0.001) (Figure 6D). The breakdown of infections is provided in Supporting Information S1: Table 4, with urinary tract infection being the most common in both groups, followed by pneumonia. In the IMS group, one patient died from staphylococcal bacteremia likely related to infective endocarditis. Furthermore, multivariate Cox regression analysis (Table 3) identified the use of IMS (HR 2.35, CI: 1.20–4.25, *p* < 0.001) and a history of chronic obstructive pulmonary disease (HR 2.00, CI: 1.22–3.27, *p* = 0.006) as significant independent predictors of infections requiring hospitalization.

**Table 3:** A multivariate Cox regression analysis on systemic infections requiring hospitalization.

	Univariable HR (95% CI)	*p* value	Multivariable HR (95% CI)	*p* value
Age (1 year increase)	1.01 (0.99–1.04)	0.379	1.02 (0.98–40.5)	0.211
Male	1.02 (0.62–1.69)	0.936	1.04 (0.50–2.15)	0.924
Diabetes mellitus	1.25 (0.77–2.03)	0.372	1.03 (0.52–2.03)	0.928
COPD	2.13 (1.31–3.49)	0.004	2.00 (1.22–3.27)	0.006
IMS	2.61 (1.45–4.69)	0.001	2.35 (1.20–4.25)	< 0.001
Prior CIED implantation	1.05 (0.61–1.64)	0.852	1.29 (0.60–2.77)	0.501

Abbreviations: CI = confidence interval, CIED = cardiac implantable electrical device, COPD = chronic obstructive pulmonary disease, HR = hazard ratio, IMS = immunosuppressive drug.

## 4 Discussion

To our knowledge, this is the first study to compare the effect of IMS therapies on outcomes in patients undergoing LAAC. The significant findings of this study are as follows: (1) There were no significant differences in implant success rates or perioperative complications for LAAC procedures in the IMS group compared to the non‐IMS group. (2) PDL and DRT at long‐term follow‐up did not differ between the two groups. However, if PDL was seen at the first evaluation, there was significantly less improvement for the patients on IMS therapies (8.3% vs. 50.0%, *p* = 0.003). Lastly, (3) the IMS group had an increased incidence of systemic infections during the follow‐up period.

### 4.1 Perioperative Complications

The relationship between steroid use and tissue fragility is well documented. Long‐term steroid administration inhibits the synthesis of granulation tissue and chondroitin sulfate, leading to increased tissue vulnerability [5, 6, 7]. In a previous large study, pericardial effusion requiring intervention was observed in 1.39%, of which 0.24% required surgical drainage and management [8]. In addition, LAAC in liver or kidney recipients showed no significant difference in interventional pericardial effusion compared without transplantation [14]. In our results, although the study population was small, LAAC with IMS can be implemented with similar risks as without.

### 4.2 Clinical Outcomes in Follow‐Up

Tacrolimus and cyclosporine are metabolized by CYP3A4, thereby inhibiting CYP3A4 and permeability glycoprotein, which increases anticoagulant potency [14]. Actually, long‐term oral administration of IMS increases the risk of infection and gastrointestinal bleeding [3, 4, 5]. Twenty‐four patients died due to infection, including one patient in the IMS group who succumbed to postoperative staphylococcal bacteremia. Although little data exists about infective endocarditis associated with LAAC devices [15], careful perioperative management is warranted. On the other hand, 62 patients (6.1%) experienced bleeding, of which 34 (3.1%) experienced bleeding 1 year after implant on aspirin therapy only. In addition, postoperative gastrointestinal bleeding was observed in 19 patients (1.9%), which was significantly higher in the IMS group than in the non‐IMS group (5.6% vs. 1.6%, *p* = 0.047), which may reflect increased tissue fragility. A possible explanation for why stroke events did not increase in the IMS group is that recent studies have reported mechanisms by which anti‐inflammatory immunosuppression can prevent thrombus formation, including the suppression of inflammatory cytokine production, inhibition of complement activation, and attenuation of platelet function [16]. These mechanisms may have contributed to the absence of an increase in DRT or ischemic events despite the limited improvement of PDL in the IMS group.

### 4.3 Changes of PDL

The reported findings of PDL have varied in the published literature, with incidence being 40.9% for the Watchman 2.5 [17], 37.0% for the Amulet [18], and 17.2% for the Watchman FLX [10]. Alkhouli et al. [12] summarized the factors associated with PDL as gender, presence of cardiomyopathy, type of atrial fibrillation, size of LAA, and displacement of the device axis. In our study, the incidence of PDL was 17.9% and 11.9% at the first and second follow‐up, respectively, and no difference was observed between those with and without IMS. Based on these results, we believe that IMS itself is not involved in the occurrence of PDL due to a tissue edge gap, off‐axis position, or device shift after implant.

In the PINNACLE FLX study, the prevalence of any PDL decreased from 17.4% at 45 days to 10.5% at 1 year [10]. In our study, the incidence of PDL in the non‐IMS group decreased from 18.4% to 11.4%, but increased from 15.2% to 18.3% in the IMS group, with significantly lower improvement in PDL severity in the IMS group. The precise mechanism underlying these changes in PDL remains unclear. However, it may be associated with delayed endothelialization or micro‐migration of the device due to left atrial remodeling [19, 20]. Afzal et al. showed that with the Watchman 2.5 device, there was less improvement in leaks over time with leaks > 3 mm versus < 3 mm “small” leaks [21]. Bhuta et al. reported that improved device fit with the Watchman FLX device improved outcomes in large leaks [22]. Our study showed no difference in the improvement of PDL size according to the initial measured severity. It has been documented that IMS can delay vascular endothelialization [23]. These drugs are often used on stents during percutaneous coronary interventions to prevent in‐stent restenosis by inhibiting endothelial proliferation [24, 25]. This inhibitory mechanism may explain the reduced PDL improvement observed in the IMS group in cases where a small leak persisted, dependent on the lack of complete surface healing.

## 5 Limitations

This study has several limitations that should be acknowledged. First, it was a single‐center, retrospective analysis. Additionally, the number of patients receiving IMS was relatively small. Device selection was not randomized, which may have introduced selection bias, as differences in device types could have affected procedural outcomes and device‐related complications. The study population was heterogeneous, and the underlying conditions necessitating IMS, as well as the types and dosages of these medications, varied and were determined at the discretion of the treating clinicians. As a result, the occurrence of adverse events may have been influenced by the specific medication or its dosage. Follow‐up imaging was performed using either TEE or CT, based on operator preference, which may have led to variability in the detection of PDL and DRT. Persistent fabric leak may indicate incomplete endothelialization. However, further studies are needed to fully elucidate the mechanisms and overall incidence of this finding.

## 6 Conclusion

LAAC is a feasible option for patients receiving IMS without a significant increase in perioperative risks. The presence of IMS in patients with PDL may result in impaired healing and leak closure.

## Conflicts of Interest

Christopher R. Ellis has received research funding from Boston Scientific, Medtronic, Boehringer Ingelheim, and consulting fees from Medtronic, Boston Scientific, Abbott Medical, and Atricure. The other authors declare no conflicts of interest.

## Supporting information


**Supplemental Table 1:** Details and dosage of immunosuppressive drugs. **Supplemental Table 2:** Comparison of characteristics and device status according to PDL change. **Supplemental Table 3:** Mechanism of residual leak. **Supplemental Table 4:** Breakdown of systemic infection.

## Data Availability

The data that support the findings of this study are available from the corresponding author upon reasonable request.
