# Supplementary material for: Impact of Immunosuppressive Drugs on Patients With Percutaneous Left Atrial Appendage Occlusion
Source: J Cardiovasc Electrophysiol. 2025 Aug 28;36(11):2979–89. doi: 10.1111/jce.70080 (PMC12614147; doi:10.1111/jce.70080)
Supplement: Supplementary file 1 — Supplemental Table 1: Details and dosage of immunosuppressive drugs. Supplemental Table 2: Comparison of characteristics and device status according to PDL change. Supplemental Table 3: Mechanism of residual leak. Supplemental Table 4: Breakdown of systemic infection. [file JCE-36-2979-s001.docx]

**Supplemental Material**

Supplemental Table 1: Details and dosage of immunosuppressive drugs.

| Drugs | n | Median dosage mg/day |
| --- | --- | --- |
| Steroid |  |  |
| Prednisone | 48 | 5 |
| Hydrocortisone | 2 | 10 |
| Non-steroids |  |  |
| Methotrexate | 8 | 15* |
| Azathioprine | 1 | 50 |
| Tacrolimus | 2 | 2 |
| Combination therapy |  |  |
| Prednisone/Methotrexate | 2 | 5/2.5* |
| Prednisone/Cyclosporine | 2 | 5/75 |
| Prednisone/Tacrolimus | 7 | 5/1 |
| Prednisone/Sirolimus | 1 | 5/0.5 |
| Prednisone/Everolimus | 1 | 5/0.75 |

*****Weekly dosage.

Supplemental Table 2: Comparison of characteristics and device status according to PDL change.

| Variable | PDL worsened or unchanged  n=89 | PDL improved  n=79 | P value |
| --- | --- | --- | --- |
| IMS | 11 (12.4) | 1 (1.3) | 0.002 |
| CHA_2_DS_2_-VASc score | 4[4-5] | 4[4-5] | 0.436 |
| HASBLED score | 3[2-3] | 3[2-3] | 0.680 |
| LAA morphology |  |  |  |
| Cauliflower | 23 (25.8) | 17 (21.5) | 0.511 |
| Chicken wing | 16 (18.0) | 16 (20.3) | 0.708 |
| Wind sock | 47 (52.8) | 42 (53.2) | 0.963 |
| Cactus | 3 (3.4) | 4 (5.1) | 0.584 |
| Type of devices |  |  |  |
| Watchman 2.5 | 38 (42.7) | 23 (29.1) | 0.074 |
| Watchman FLX | 35 (39.3) | 40 (50.6) | 0.141 |
| Amulet | 16 (18.0) | 16 (20.3) | 0.708 |
| LAA ostium diameter, mm | 22[19.5-24.8] | 22[20-24] | 0.901 |
| LAA depth, mm | 30[27-34] | 30[24-31] | 0.057 |
| Intraoperative LAP, mmHg | 12[8-14] | 11[7-15] | 0.942 |
| Compression rate (watchman), % | 20[19.3-25] | 20[20-25.3] | 0.488 |
| Number of recapture, times | 1[1-1] | 1[1-1] | 0.721 |
| PDL at 1st follow-up |  |  |  |
| Minor | 53 (59.6) | 52 (65.8) | 0.401 |
| Moderate | 25 (28.1) | 19 (24.1) | 0.552 |
| Major | 3 (3.4) | 8 (10.1) | 0.074 |
| Stroke after LAAC | 3 (3.4) | 3 (3.8) | 0.882 |

Continuous variables are displayed as the median[Q1-Q3].

Categorical variables are presented as mean ± standard deviation or n(%).

IMS = immunosuppressive drug, LAA = left atrial appendage, LAAC = left atrial appendage closure, LAP = left atrial pressure, PDL = peri-device leak.

Supplemental Table 3: Mechanism of residual leak.

|  | Watchman 2.5  n=66 | Watchman FLX  n=80 | Amulet  n=33 |
| --- | --- | --- | --- |
| Peri-device leak | 61 (92.4) | 75 (93.8) | 32 (97.0) |
| Proximal placement or from the ridge side | 17 (25.8) | 13 (16.3) | N/A |
| Distal placement or Mis-lobed | 2 (3.0) | 9 (11.3) | N/A |
| Uncovering due to the pectinate muscle | 14 (21.2) | 15 (18.8) | N/A |
| Off axis | 0 (0) | 5 (6.3) | N/A |
| Edge leak | 28 (42.4) | 33 (41.3) | N/A |
| Leak between disc and lobe | N/A | N/A | 16 (48.5) |
| Leak into distal lobe | N/A | N/A | 16 (48.5) |
| Fabric leak | 5 (7.6) | 5 (6.3) | 1 (3.0) |

Values are presented as n(%).

Supplemental Table 4: Breakdown of systemic infection

|  | Total  n=73 | |
| --- | --- | --- |
|  | IMS group  n=18 | Non-IMS group  n=55 |
| Pneumonia | 5(27.8) | 18(32.7) |
| Urinary tract infection | 9(50.0) | 21(38.2) |
| Infective endocarditis | 2(11.1) | 1(1.8) |
| **Cellulitis** | 2(11.1) | 4(7.3) |
| **Gastroenteritis,** C**holecystitis, Diverticulitis** | 0(0) | 5(9.1) |
| **Unknown focus** | 0(0) | 6(7.3) |

Values are presented as n(%).
